# A comparative study of clinical efficacy, electrophysiological outcomes, and perioperative parameters between endoscopic carpal tunnel release and open carpal tunnel release for carpal tunnel syndrome

**DOI:** 10.3389/fncel.2026.1845917

**Published:** 2026-06-03

**Authors:** Zhiqi Zhang, Yulong Liu, Huarui Yang, Bihui Song, Kangquan Shou

**Affiliations:** Department of Orthopedics, The First College of Clinical Medical Sciences, China Three Gorges University and Yichang Central People’s Hospital, Yichang, China

**Keywords:** carpal tunnel release, carpal tunnel syndrome, endoscopy, neurophysiology, treatment outcome

## Abstract

**Purpose:**

To compare the clinical efficacy of endoscopic (ECTR) and open (OCTR) carpal tunnel release for carpal tunnel syndrome (CTS) and provide evidence-based surgical guidance.

**Methods:**

A retrospective cohort study was conducted including 112 consecutive patients with CTS treated at our institution between January 2022 and December 2024. Based on the surgical approach, patients were assigned to an ECTR group or an OCTR group. Surgical selection was determined by patients’ preference, cosmetic expectations, and the surgeon’s technical assessment. Primary outcome measures were the Boston Carpal Tunnel Questionnaire Symptom Severity Score (BCTQ-SSS), Functional Status Score (BCTQ-FSS), and pain assessed by the Visual Analog Scale (VAS). Secondary outcome measures included median nerve electrophysiological indices [sensory nerve conduction velocity (SNCV), sensory nerve action potential (SNAP) amplitude, distal motor latency (DML), and compound muscle action potential (CMAP) amplitude], operative time, hospitalization cost, and postoperative complications. All outcomes were evaluated preoperatively and at the final follow-up (cutoff date: February 2026, with postoperative follow-up duration ranging from 13 to 48 months).

**Results:**

Eighty-two eligible patients were followed up for a median of 34 months (13–48 months). Both groups showed significant improvements in VAS, BCTQ scores, and electrophysiological parameters versus baseline (all *P* < 0.05), with no intergroup differences (all *P* > 0.05). OCTR had significantly shorter operative time and lower cost (both *P* < 0.001), and complication rates did not differ significantly (*P* > 0.05).

**Conclusion:**

ECTR and OCTR have comparable mid-term efficacy for CTS. OCTR is more cost-effective with shorter operative time, while ECTR’s minimally invasive advantages need further confirmation. Surgical choice should be individualized based on patient preferences and institutional resources.

## 1 Introduction

Carpal tunnel syndrome (CTS) is the most prevalent upper extremity peripheral nerve entrapment neuropathy worldwide, with a prevalence of 3.8% in the general population. In the United States alone, over 600,000 patients undergo carpal tunnel release (CTR) annually, imposing a substantial burden on healthcare systems and societal workforce productivity (Latzka et al., 2018; Pripotnev and Mackinnon, 2022; Cognet et al., 2025). As the leading cause of hand dysfunction secondary to nerve entrapment, CTS and its standardized surgical management have long been a key research focus and clinical hotspot in the field of hand surgery.

The core pathological change of CTS is compression of the median nerve within the carpal tunnel, which can lead to progressive pain, paresthesia, and motor dysfunction of the hand, severely impairing patients’ quality of life and work capacity (Xu J. et al., 2025). For patients with moderate-to-severe CTS who show no response to conservative management, surgical division of the transverse carpal ligament (TCL) to achieve median nerve decompression is currently a well-recognized definitive treatment strategy (Bland, 2007). Conventional open carpal tunnel release (OCTR) has long been regarded as the gold standard procedure for CTR, with the advantages of adequate surgical field exposure, a low technical threshold, and broad clinical indications (Wellborn et al., 2025; Boutros et al., 2026). As a minimally invasive alternative, endoscopic carpal tunnel release (ECTR) has seen a continuous rise in clinical adoption since its introduction. Its theoretical benefits include minimal soft tissue trauma and a more cosmetically favorable postoperative scar, theoretically enabling a more rapid postoperative recovery (MacDermid et al., 2025; Xu J. et al., 2025; Xu Y. et al., 2025; Zheng et al., 2026).

Previous comparative studies and systematic reviews evaluating these two surgical approaches have yielded inconsistent conclusions (Dondapati et al., 2025). In a randomized controlled trial, MacDermid et al. (2025) found that ECTR conferred a short-term advantage in grip strength recovery and pain relief at 1 and 6 weeks postoperatively, but this difference was completely abolished by 12 weeks after surgery. The latest meta-analysis by Boutros et al. (2026), which enrolled 44 comparative studies, revealed no statistically significant differences between the two techniques in terms of long-term pain relief, functional recovery, muscle strength improvement, and overall complication rates, while the ECTR group had a significantly lower postoperative revision rate. However, most existing studies have focused on Western populations, with limited real-world evidence from Chinese clinical settings, and few studies have simultaneously compared clinical efficacy, electrophysiological outcomes, and perioperative economic parameters of the two approaches.

To further clarify the clinical application value of different CTR surgical techniques, we conducted a single-center retrospective cohort study. We proposed the following hypothesis: there is no significant difference in the mid-term clinical efficacy of ECTR and OCTR in the treatment of CTS, while there are significant differences in perioperative parameters between the two approaches. We collected the clinical data and follow-up information of patients with CTS who underwent surgical treatment at our institution, and performed a comparative analysis of the clinical efficacy, median nerve electrophysiological outcomes, perioperative parameters, and complications between OCTR and ECTR. This study aims to provide real-world evidence from Chinese clinical settings to support individualized clinical decision-making in the selection of surgical approaches for CTS.

## 2 Materials and methods

This study was approved by the Ethics Committee of Yichang Central People’s Hospital (approval number: 2026-021-01) and performed in strict accordance with the Declaration of Helsinki. All enrolled patients signed informed consent for their clinical data to be used in this retrospective study.

### 2.1 Inclusion and exclusion criteria

This was a single-center retrospective cohort study. We consecutively enrolled patients with CTS who underwent surgical treatment (OCTR or ECTR) at the Department of Orthopedics, Yichang Central People’s Hospital from January 2022 to December 2024.

The inclusion criteria for this study are as follows: (1) Confirmed clinical diagnosis of primary carpal tunnel syndrome (CTS) based on characteristic symptoms and standardized physical examination findings; (2) Electrophysiological confirmation of CTS according to established diagnostic criteria, characterized by prolonged distal motor latency (DML), reduced compound muscle action potential (CMAP) amplitude, prolonged sensory latency, reduced sensory nerve action potential (SNAP) amplitude, slowed sensory nerve conduction velocity (SNCV), and absent motor/sensory potentials in severe cases (Chong et al., 2024); (3) Failure of a standardized 2-month conservative treatment regimen, including wrist brace immobilization, oral neurotrophic medications, and local physical therapy; (4) Complete clinical records and a minimum follow-up duration of 6 months.

The exclusion criteria were as follows: (1) CTS cases in which surgical treatment was not performed according to the study protocol; (2) Secondary CTS caused by space-occupying lesions within the carpal tunnel confirmed by preoperative ultrasonography; (3) Bony or articular abnormalities of the carpal tunnel identified on plain radiography; (4) Concurrent wrist osteoarthritis, rheumatoid arthritis, or gouty arthritis.

All enrolled patients underwent preoperative wrist plain radiography and high-frequency ultrasonography to rule out space-occupying lesions and bony abnormalities of the carpal tunnel, which was a mandatory preoperative examination procedure in our institution.

### 2.2 Clinical data

**FIGURE 1:**
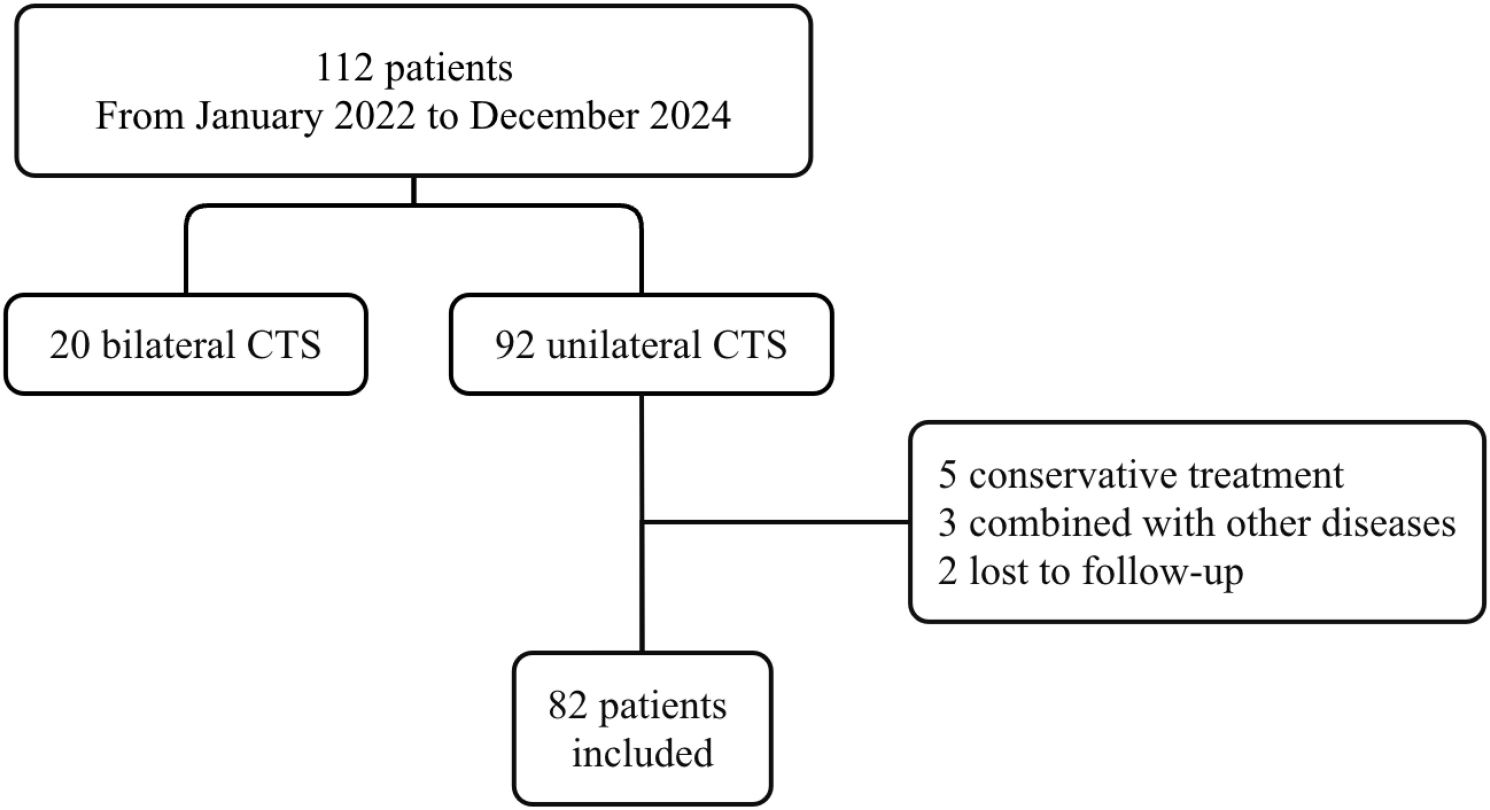
Flow chart showing patient cohort inclusion.

Between January 2022 and December 2024, a total of 112 patients were diagnosed with CTS. Of these, 82 patients who met the inclusion criteria were enrolled in this study (Figure 1). A comprehensive retrospective review was performed on the clinical data and electrophysiological characteristics of the enrolled patients.

Baseline clinical data included age, sex, and body mass index (BMI). Primary outcome measures comprised the Boston Carpal Tunnel Questionnaire Symptom Severity Score (BCTQ-SSS), Boston Carpal Tunnel Questionnaire Functional Status Score (BCTQ-FSS), and pain scores measured using the Visual Analog Scale (VAS). All primary outcomes were evaluated at two time points: preoperatively and at the final follow-up visit.

Secondary outcome measures consisted of the following: (1) Median nerve electrophysiological parameters, including sensory nerve conduction velocity (SNCV), sensory nerve action potential (SNAP) amplitude, distal motor latency (DML), and compound muscle action potential (CMAP) amplitude. These parameters were evaluated by the same experienced neurophysiologist preoperatively and at the final follow-up visit using a standardized electromyography system. (2) Perioperative parameters: operative time was defined as the time elapsed from skin incision to the completion of skin closure; total hospitalization cost included surgical fees, anesthesia fees, implant and equipment fees, as well as hospitalization and nursing fees, which were converted to US dollars at an exchange rate of 1 USD = 7.2 Chinese Yuan at the time of patient discharge. (3) Postoperative complications, including wound infection, nerve injury, vascular injury, symptom recurrence, and reoperation. All patients were followed up for a minimum of 12 months to assess the incidence of postoperative complications. Complication monitoring was performed according to a standardized protocol: routine outpatient follow-ups at 1 week, 1 month, 3 months, and 6 months postoperatively, followed by telephone or outpatient follow-ups every 6 months thereafter.

All questionnaires were completed independently by each patient under the guidance of a specialized nurse, without any clinical intervention or prompting during completion to guarantee the authenticity and objectivity of the collected data.

### 2.3 Boston carpal tunnel questionnaire

The Boston Carpal Tunnel Questionnaire (BCTQ) was developed by Levine et al. (1993), and the Chinese version of BCTQ used in this study has been validated in Chinese CTS patients, with good reliability and validity (Lue et al., 2014). It consists of two core domains: the Symptom Severity Score (SSS) and the Functional Status Score (FSS). The SSS evaluates the frequency and severity of core CTS symptoms, while the FSS assesses the impact of CTS on patients’ ability to perform activities of daily living (ADLs). The clinically recognized thresholds for the Patient Acceptable Symptom State (PASS) are an SSS ≤ 1.82 and an FSS ≤ 1.50; scores below these thresholds indicate that the patient has achieved clinically satisfactory recovery of symptoms and functional status (Maughan et al., 2025).

### 2.4 Neuroelectrophysiology examination

The 2025 updated Clinical Practice Guideline for CTS issued by the American Academy of Orthopedic Surgeons (AAOS) has identified neuroelectrophysiological assessment as a core reference modality for CTS diagnosis, severity stratification, and prognostic evaluation (Shapiro and Kamal, 2025). The median nerve SNCV, SNAP amplitude, DML, and CMAP amplitude were used as the core electrophysiological parameters in this study, with standardized detection and measurement protocols consistent with the guideline recommendations (Padua et al., 2016).

### 2.5 Statistical analysis

Statistical analyses were performed using SPSS version 25.0 software (SPSS Inc., Chicago, IL, United States). Categorical variables were presented as frequencies and percentages, and intergroup comparisons were conducted using the chi-square test. Continuous variables were expressed as mean ± standard deviation (SD). The Shapiro-Wilk test was used to verify the normality of data distribution, and Levene’s test was applied to assess the homogeneity of variances between groups. For intergroup comparisons of continuous variables, the independent samples *t*-test was used when data were normally distributed with homogeneous variances, while Welch’s corrected *t*-test was adopted in cases of heterogeneous variances. The Mann-Whitney U test was performed for data with a non-normal distribution. A *P*-value < 0.05 was considered to indicate a statistically significant difference.

## 3 Surgical procedure

OCTR was performed using the mini-palm open release technique, which was first described by Bromley (1994). ECTR was conducted with a specialized technique using a transparent, colorless plastic protective sheath fabricated from a syringe, which was first reported by Liu and Wu (2021). The choice of surgical approach was jointly determined by the attending surgeon and the patient before surgery. The main selection criteria included: (1) Patients with high cosmetic needs and willingness to accept minimally invasive surgery were recommended for ECTR; (2) Patients with cost sensitivity or who were not suitable for endoscopic surgery due to anatomical variations were recommended for OCTR. The main anatomical variations precluding ECTR include aberrant course of the palmar cutaneous branch of the median nerve, persistent median artery, hypertrophic and calcified transverse carpal ligament, and bony prominences of the carpal bones, which increase the risk of neurovascular injury during endoscopic procedures; (3) All surgeries were performed by the same group of surgeons with more than 5 years of experience in hand surgery, to ensure the consistency of surgical proficiency.

## 4 Results

Between January 2022 and December 2024, a total of 112 patients were diagnosed with CTS. Of these, 82 patients (55 females and 27 males) met the inclusion criteria and were enrolled in this study. The median follow-up duration of all patients was 34 months (range, 13–48 months). Subgroup analysis showed that the median follow-up duration was 33 months (range, 13–48 months) in the OCTR group and 35 months (range, 14–47 months) in the ECTR group, with no statistically significant difference between the two groups (*P* = 0.682). Among the enrolled patients, 47 cases underwent OCTR, and the remaining 35 cases received ECTR. The detailed patient enrollment flowchart is shown in Figure 1, with specific reasons for exclusion: 12 patients had bilateral CTS, 8 patients had a follow-up period <6 months, 5 patients had carpal tunnel space-occupying lesions, 3 patients had wrist joint inflammatory arthritis, and 2 patients had incomplete clinical data.

We first compared the preoperative baseline characteristics between the two groups of patients. There were no statistically significant differences in age, sex distribution, and body mass index (BMI) between the two groups (all *P* > 0.05), indicating that the demographic characteristics of the two groups were comparable (Table 1). No statistically significant differences were observed in the preoperative VAS score, BCTQ-SSS, and BCTQ-FSS between the OCTR group and the ECTR group (all *P* > 0.05). For the preoperative neuroelectrophysiological parameters, including SNCV, SNAP amplitude, DML, and CMAP amplitude, no significant differences were detected between the two groups (all *P* > 0.05, Table 2). These results confirmed that the baseline characteristics of the two groups were well-balanced and comparable.

**TABLE 1:** Patient demographics characteristics.

Variables	Open (*n* = 47)	Endoscopic (*n* = 35)	*P-*value
Age (years)	52.353 ± 9.08	54.89 ± 12.01	0.279
Sex (M/F)	15 (31.91)/32 (68.08%)	12 (35.29%)/23 (65.71%)	0.821
BMI (kg/m^2^)	23.65 ± 2.87	24.32 ± 2.49	0.272
Left/right	19 (40.43%)/28 (59.57%)	13 (37.14%)/22 (62.86%)	0.763
Follow-up duration (months)	33 (range, 13–48)	35 (range, 14–47)	0.682

BMI, body mass index.

**TABLE 2:** Preoperative and postoperative VAS scores, BCTQ scores, and nerve electrophysiological examination outcomes of patients.

Variables	Prior to surgery			Final follow-up		
	Open (*n* = 47)	Endoscopic (*n* = 35)	*P-*value	Open (*n* = 47)	Endoscopic (*n* = 35)	*P-*value
VAS	2.87 ± 0.82	3.00 ± 1.10	0.558	0.38 ± 0.49	0.34 ± 0.48	0.713
BCTQ-SSS	32.47 ± 5.52	34.43 ± 6.04	0.136	12.60 ± 1.16	12.69 ± 1.23	0.735
BCTQ-FSS	21.53 ± 4.18	20.83 + 5.66	0.539	9.62 ± 1.47	9.89 ± 1.30	0.390
SNCV (m/s)	39.12 ± 3.99	39.49 ± 2.23	0.623	45.19 ± 2.59	45.35 ± 2.94	0.795
SNAP (μ V)	12.03 ± 1.87	12.41 ± 1.75	0.348	15.88 ± 3.90	16.05 ± 2.14	0.816
DML (ms)	5.23 ± 0.34	5.14 ± 0.32	0.227	4.80 ± 0.47	4.77 ± 0.42	0.766
CMAP (mV)	9.30 ± 1.41	9.76 ± 1.30	0.130	12.07 ± 1.18	12.31 ± 2.46	0.560

BCTQ-SSS, the symptom severity scale of Boston carpal tunnel questionnaire; BCTQ-FSS, the functional status scale of Boston carpal tunnel questionnaire; CMAP, compound muscle action potential; DML, distal motor latency; SNAP, sensory nerve action potential; SNCV, sensory nerve conduction velocity.

At the final follow-up, both groups achieved remarkable improvements in clinical symptom scores and nerve electrophysiological indicators relative to their preoperative baseline levels (all *P* < 0.05). In the intergroup comparison, no statistically significant differences were found in the VAS scores (0.38 ± 0.49 vs. 0.34 ± 0.48, *P* = 0.713), BCTQ-SSS scores (12.60 ± 1.16 vs. 12.69 ± 1.23, *P* = 0.735), and BCTQ-FSS scores (9.62 ± 1.47 vs. 9.89 ± 1.30, *P* = 0.390) between the OCTR and ECTR groups. Consistently, the final follow-up nerve electrophysiological outcomes, including SNCV (45.19 ± 2.59 m/s vs. 45.35 ± 2.94 m/s, *P* = 0.795), SNAP (15.88 ± 3.90 μV vs. 16.05 ± 2.17 μV, *P* = 0.816), DML (4.80 ± 0.47 ms vs. 4.77 ± 0.42 ms, *P* = 0.766), and CMAP (12.07 ± 1.18 mV vs. 12.31 ± 2.46 mV, *P* = 0.560), showed no significant differences between the two groups (all *P* > 0.05, Table 2).

The perioperative outcomes of the two groups are summarized in Table 3 (Detailed data can be found in the Supplementary material). The mean operative time was significantly longer in the ECTR group than in the OCTR group (47.17 ± 5.54 min vs. 35.25 ± 5.16 min, *P* < 0.001). In addition, the total hospitalization cost of the ECTR group was significantly higher than that of the OCTR group [7756.86 ± 691.26 Yuan (1077.34 ± 96.01 USD) vs. 6516.58 ± 430.60 Yuan (905.08 ± 59.81 USD), *P* < 0.001].

**TABLE 3:** Perioperative outcomes of the two groups.

Variables	Open (*n* = 47)	Endoscopic (*n* = 35)	*P-*value
Operative time (min)	35.25 ± 5.16	47.17 ± 5.54	< 0.001
Costs (Yuan)	6516.58 ± 430.60	7756.86 ± 691.26	< 0.001
Cost (USD)	905.08 ± 59.81	1077.34 ± 96.01	< 0.001

During the follow-up period, no major complications such as median nerve injury, vascular injury, or tendon injury occurred in either group. In the OCTR group, 2 cases (4.26%) had superficial wound infection, which healed after dressing change and antibiotic treatment; no symptom recurrence or reoperation occurred. In the ECTR group, 1 case (2.86%) had transient paresthesia of the median nerve, which resolved within 2 weeks after conservative treatment; no symptom recurrence or reoperation occurred. There was no significant difference in the overall incidence of postoperative complications between the two groups (4.26% vs. 2.86%, *P* = 0.925).

## 5 Discussion

In this single-center retrospective analysis enrolling 82 patients with CTS, we compared the clinical efficacy, perioperative parameters, and long-term follow-up outcomes between ECTR and OCTR. The core finding was that there were no statistically significant differences between the two surgical approaches in terms of postoperative pain relief, functional recovery, and neuroelectrophysiological improvement, whereas OCTR exhibited significant advantages of shorter operative time and lower hospitalization costs. These findings not only fulfill the study objective of clarifying the clinical application value of the two CTR modalities stated in the Introduction but also provide real-world evidence-based support for clinical surgical decision-making in CTS. Meanwhile, the clinical implications and scientific value of these results warrant in-depth interpretation in the context of the existing research landscape and the inherent limitations of the present study.

CTR is the first-line definitive treatment for moderate to severe CTS after failure of standardized conservative treatment, and the optimization of surgical modalities has long been a key research focus in hand surgery (Kim et al., 2025). The results of this study showed that both groups achieved significant improvements in VAS score, BCTQ-SSS, BCTQ-FSS, and all median nerve electrophysiological parameters after surgery, with no significant intergroup differences in these efficacy outcomes. This finding is consistent with the meta-analysis by El Masri et al. (2024), which confirmed no significant differences in long-term pain relief, functional recovery, and overall complication rate between the two surgical modalities, further verifying the reliable clinical efficacy of both CTR procedures. The RCT by MacDermid et al. (2025) showed that ECTR had a short-term advantage in pain relief and grip strength recovery at 1–6 weeks postoperatively, but this difference disappeared at 12 weeks after surgery, which is highly consistent with the mid-term follow-up results (median 34 months) of this study, confirming that the two approaches have comparable mid- to long-term efficacy. Furthermore, neuroelectrophysiological parameters are recognized as the core benchmarks for evaluating postoperative recovery in CTS.

In this study, we used median nerve electrophysiological parameters as one of the core outcome measures, which is consistent with the recommendations of the AAOS CTS clinical practice guideline (Shapiro and Kamal, 2025). However, it should be noted that existing studies have different views on the correlation between electrophysiological examination results and postoperative symptomatic outcomes. A systematic review by Osiak et al. (2021) pointed out that although electrodiagnostic (EDX) examination is a valuable diagnostic tool for CTS, it is not essential for outcome assessment in patients with clear clinical diagnosis of CTS. Meanwhile, Glowacki et al. (1996) found that electrodiagnostic testing results did not correlate with the final symptomatic outcome after CTR in patients with clinically definite CTS. In clinical practice, electrophysiological examination may cause varying degrees of discomfort and even pain in some patients. Therefore, for patients with clear clinical diagnosis of CTS and no need for severity stratification, whether routine postoperative electrophysiological reexamination is necessary still needs to be further verified by prospective studies.

About perioperative parameters, the present study revealed that OCTR was associated with a significantly shorter operative time and significantly lower hospitalization costs compared with ECTR (both *P* < 0.001). This finding aligns with the conclusions of a portion of prior studies, while conflicting with those reported in other published investigations. Studies by Teng et al. (2019) and Zheng et al. (2023) reported that endoscopic carpal tunnel release had a shorter operative time compared with open surgery. Such discrepancies in study findings may be attributed to variations in surgical modality selection, surgeon operative proficiency, and the severity of patients’ CTS. Notably, in the present study, the ECTR procedure was performed using a novel endoscopic technique, which required preparation of a transparent plastic protective sheath fabricated from a standard syringe. In contrast, the modified mini-palm open release technique adopted for OCTR provided adequate surgical field exposure and a simplified operative workflow, thus resulting in a shorter operative duration. A 2024 study by Thomas et al. (2024) also reported that conventional ECTR generally required a longer operative time than conventional open surgery, as it necessitated the establishment of an endoscopic working channel and precise anatomical localization of carpal tunnel structures. Meanwhile, the utilization of endoscopic equipment was associated with increased hospitalization costs, which is consistent with the finding of higher hospitalization costs for ECTR in the present study.

The core mechanism of clinical efficacy of both surgical approaches is complete release of the TCL, which relieves mechanical compression of the median nerve, restores nerve blood supply and conduction function, and thus improves clinical symptoms. The significant improvement in electrophysiological parameters and BCTQ scores in both groups after surgery confirmed that both approaches can achieve adequate decompression of the median nerve, which is consistent with the conclusions of Alrayes et al. (2024). The Chinese version of BCTQ used in this study has been validated in Chinese CTS patients, ensuring the reliability and validity of the symptom and function assessment results (Lue et al., 2014).

The clinical value of the present study mainly lies in providing precise evidence-based reference for surgical decision-making of CTS in real-world clinical settings. As the most prevalent upper extremity peripheral nerve entrapment disorder worldwide, CTS has a prevalence rate of 9.6% among office workers in China, has been included in China’s statutory list of occupational diseases, and a large number of patients undergo CTR annually (Feng et al., 2021). Selecting a more cost-effective and efficient surgical modality while ensuring consistent therapeutic efficacy is of great clinical and socioeconomic significance for alleviating the healthcare burden and improving patients’ healthcare experience. The present study demonstrated that OCTR and ECTR achieved comparable clinical efficacy, whereas OCTR exhibited distinct advantages in operative time and medical costs, making it particularly suitable for primary healthcare institutions and cost-sensitive patients. As a minimally invasive surgical modality, ECTR has the theoretical advantages of less soft tissue trauma and better cosmetic appearance, which may be a priority consideration for patients with relevant needs, but this needs to be verified by further quantitative studies on scar assessment and postoperative recovery. These findings are consistent with the results reported by Rajapandian et al. (2024), which stated that the cosmetic advantage of ECTR represents a core value for its clinical application, while the high efficiency and cost-effectiveness of OCTR are more favorable for large-scale clinical implementation. The main innovation of this study is that it provides real-world clinical evidence from the Chinese population, and simultaneously compares the clinical efficacy, electrophysiological outcomes, and perioperative economic parameters of the two surgical approaches, which complements the existing research gap. The results of this study can provide a direct reference for individualized surgical decision-making in clinical practice and also provide a basis for the selection of surgical modalities in primary medical institutions.

Several limitations of the present study should be objectively acknowledged. First, this study was a single-center retrospective analysis with a limited enrolled sample size of 82 patients. As a retrospective observational study, it carries inherent limitations including potential selection bias and information bias, and its level of evidence is lower than that of prospective randomized controlled trials (RCTs). The relatively small sample size may lead to insufficient statistical power to detect minor but clinically significant differences between the two surgical approaches. Second, the surgical approach allocation in this study was not randomized but based on joint decision-making between surgeons and patients, considering factors such as patient preference, cosmetic expectations, and anatomical variations. Although no statistically significant differences were observed in all baseline demographic, clinical, and electrophysiological characteristics between the two groups, potential unmeasured confounding factors may still exist, which could affect the internal validity of the study results. Third, only patients with unilateral CTS were included, which inevitably restricts the generalizability of the findings. The results cannot be extrapolated to complex clinical scenarios, including patients with bilateral CTS, secondary CTS caused by space-occupying lesions, or those complicated with other wrist joint disorders. Fourth, the median follow-up duration of this study was 34 months. Although this meets the standard for medium-to-long-term follow-up, it remains insufficient to evaluate the long-term efficacy and complication risk of the two surgical modalities beyond 5 years postoperatively. Previous studies have indicated that a minimum follow-up period of 5 years is required for patients after CTS surgery to assess the long-term stability of median nerve function and the risk of reoperation (Kahan et al., 2025). Fifth, no standardized and unified postoperative rehabilitation protocol was implemented for the enrolled patients in the present study, while rehabilitation interventions have a well-recognized critical impact on postoperative functional recovery after CTS surgery (Newington et al., 2022). This may act as a potential confounding factor influencing the differences in functional recovery between the two groups. Finally, no blinded assessment of outcome measures was performed in the present study, which may introduce subjective bias; this is a well-recognized common limitation inherent to retrospective study designs. Multicenter, large-sample, prospective randomized controlled trials (RCTs) are warranted in the future to further validate the findings and conclusions of the present study.

## 6 Conclusion

This single-center retrospective cohort study confirms that both ECTR and OCTR have favorable and comparable mid-term clinical efficacy in the treatment of CTS, with no significant differences in symptom relief, functional recovery, median nerve electrophysiological improvement, and overall complication rate between the two approaches. OCTR has significant advantages of shorter operative time and lower hospitalization cost, while ECTR has the theoretical advantage of minimally invasive soft tissue injury. In clinical practice, the choice of surgical approach can be tailored to the patient’s individual needs, the technical capabilities of the medical institution, and the surgeon’s operative experience.

## Data Availability

The original contributions presented in this study are included in this article/Supplementary material, further inquiries can be directed to the corresponding authors.
